# Factors influencing the free maternal health care policy under the national health insurance scheme’s provision for skilled delivery services in Ghana: a narrative literature review

**DOI:** 10.1186/s12884-023-05730-2

**Published:** 2023-06-14

**Authors:** Juliet Abredu, Boo Alipitio, Catherine K. Dwumfour, Sophie Witter, Veronica Millicent Dzomeku

**Affiliations:** 1 Ho Nurses’ Training College, Ho, Ghana; 2 Department of Nursing, College of Health Sciences, Kwame Nkrumah University of Science and Technology, Kumasi, Ghana; 3 Institute for Global Health and Development, Queen Margaret University, Musselburgh, UK

**Keywords:** Skilled birth attendant/attendance, Facility delivery, Free maternal Health Care Policy, National Health Insurance Scheme, Ghana.

## Abstract

**Background:**

Skilled Birth Attendance (SBA) is important in achieving the Sustainable Development Goals (SDGs) targets 3.1, 3.2 and 3.3.1. Ghana has made steady progress in SBA, yet, unsupervised deliveries still occur. The introduction of the Free Maternal Health Care Policy under the National Health Insurance Scheme (FMHCP under the NHIS) has improved the uptake of SBA but with some implementation challenges. This narrative review sought to explore the factors influencing the FMHCP under the NHIS provision for skilled delivery services in Ghana.

**Methods:**

Electronic searches were conducted of databases including PubMed, Popline, Science direct, BioMed Central, Scopus and Google scholar for peer reviewed articles as well as grey articles from other relevant sources, published between 2003 and 2021 on factors influencing FMHCP/NHIS provision for skilled delivery services in Ghana. Keywords used in the literature search were in various combinations for the different databases. The articles were screened to determine the inclusion and exclusion criteria and quality was assessed using a published critical appraisal checklist. A total of 516 articles were retrieved for initial screening based on their titles, of which 61 of them, were further screened by reading their abstracts and full text. Of this number, 22 peer-reviewed and 4 grey articles were selected for the final review based on their relevance.

**Results:**

The study revealed that the FMHCP under the NHIS does not cover the full costs associated with skilled delivery and low socioeconomic status of households affects SBA. Also, funding and sustainability, hinders the quality-of-service delivery offered by the policy.

**Conclusion:**

For Ghana to achieve the SDGs above and further improve SBA, the cost associated with skilled delivery should be fully covered by the NHIS. Also, the government and the key stakeholders involved in the policy implementation, must put in place measures that will enhance the operation and the financial sustainability of the policy.

## Background

Worldwide, about 810 women die every day from pregnancy-related and delivery complications. Although maternal mortality has seen a decline from 342 deaths per 100,000 to 211 deaths per 100,000 from 2000 to 2017 respectively, Sub-Saharan Africa and Asia still recorded 86% of all the deaths revealing the wide disparity across regions [1]. This implies that, the possibility of a woman dying due to pregnancy and delivery complications in low-income countries is 33 times more than that of her counterpart in the developed countries [2, 3]. Ghana has made steady progress in the reduction of Maternal Mortality Ratio (MMR) from 760 to 100,000 live births to 350 per 100,000 between 1990 and 2010 [1]. The current MMR is 310 per 100,000 livebirths [4]. The Neonatal Mortality Ratio (NMR) has also decreased slightly from 43/1000 live births to 30/1000 live births and then increased again to 32/1000 live births in 2003, 2008 and 2011 respectively [5]. Currently, Ghana’s NMR is 25/1000 live births [4].

Skilled attendance at birth, improved health facility delivery and family planning are key strategies that reduce maternal and neonatal morbidity and deaths. However, the proportion of births attended by skilled professionals is still below the recommended target particularly in sub-Saharan Africa where only about half of births are delivered by Skilled Birth Attendants (SBAs)[6, 7]. In Ghana, there has been a steady increase in SBA from 44% to 1993 to 68% in 2011 [1]. According to the 2017 Ghana Maternal Health Survey, the proportion of births attended by SBAs in Ghana is 79% [4]. This progress is attained after the implementation of the FMHCP/NHIS.

The FMHCP was introduced by the Ghana government in 2003 with the aim of reducing financial barriers to accessing delivery services and to reduce the maternal mortality rate [8]. The policy was first implemented in the four most deprived regions of the country (Northern, Upper West (UW/R), Upper east (UE/R) and Central regions), and later extended to the six remaining regions in 2005 [8, 9]. In 2008, the FMHCP was integrated into the already existing NHIS which had started in 2005. This FMHCP under the NHIS is a full maternal benefit package which covers six free antenatal visits; emergency outpatient visits; free facility delivery including all complicated deliveries and two postnatal visits within six weeks; waives the premium on the NHIS as well as the registration fees, and covers the pregnant woman for a year, as well as the newborn baby for the first three months of life [10, 11]. The pregnant women have access to these services directly and not through family membership or their male partners. To be eligible for the full maternal package, the pregnant woman would have to provide evidence of pregnancy (pregnancy test result) from a health facility. Pregnant women who have not subscribed previously on the scheme or do not have valid cards, would also need to show evidence of pregnancy from a health facility to the NHIS service providers in order to be exempted from the NHIS registration/premium fees and have access to the ‘free’ services (*Authors statement*).

The aim of the implementation of the FMHCP under the NHIS as well as the Community-based Health Planning and Services (CHPS) strategies is to improve access to skilled delivery in Ghana [1]. However, the SBA rate is still less than the global target of 90% and the national target of 80% [12].

In ensuring that Ghana makes good progress towards decreasing maternal deaths, ending preventable new born deaths and reducing new HIV infections as proposed by the SDGs 3.1, 3.2 and 3.3.1 respectively, efforts must be scaled up towards effective uptake of skilled birth utilization and the provision of quality skilled delivery services, to make it universally possible as advocated by the World Health Organization (WHO) [13].

It is therefore expedient to explore the influence of the FMHCP under the NHIS policy-related factors on skilled delivery services in Ghana. This study will help to understand the main hindrances and gaps in the implementation of the policy and propose potential solutions that will effectively address the issue of bringing the residual non-users on board.

## Methods

### Study design

This research was a narrative review of literature of factors influencing The FMHCP under the NHIS provision for skilled delivery services in Ghana.

### Research questions

The research questions addressed were:

1. What is the extent of coverage of the NHIS and its impact on skilled delivery?
2. How does the implementation of the FMHCP under the NHIS impact skilled delivery services?
3. What policy interventions should be put in place to improve the successful operation of the FMHCP under the NHIS to achieve the WHO SBA target in Ghana?

### Search strategy

To improve the chances of identifying all relevant literature, an extensive electronic search was done from the databases including PubMed, Science direct, Scopus, Popline, BioMed Central, google scholar, the webpages of National Health Insurance Authority (NHIA), and the World Bank. The keywords used for the search were: skilled birth utilization, skilled birth attendant/attendance, facility delivery, home delivery, maternal mortality, Free Maternal Health Care Policy, National Health Insurance Scheme, and Ghana. These terms were used in combination of terms such as ‘predictors’ or ‘determinants’ or ‘factors influencing’ and ‘Ghana’. Hand-searching of relevant references from included studies was also done and the full text of articles retrieved. Further, the guidance of scholars in the field was sought, for the right source of scholarly materials to be included.

### Inclusion criteria

Peer reviewed and grey literature written in English Language and were published between 2003 and 2021 were included as the NHIS was introduced in 2003. Articles explicitly focused on Ghana including both descriptive and evaluative studies, were included in the study.

### Exclusion criteria

Articles not written in English language as well as articles with dates earlier than 2003 and did not meet the required inclusion criteria were not included in the study.

### Selection and exclusion of articles

**Fig. 1:**
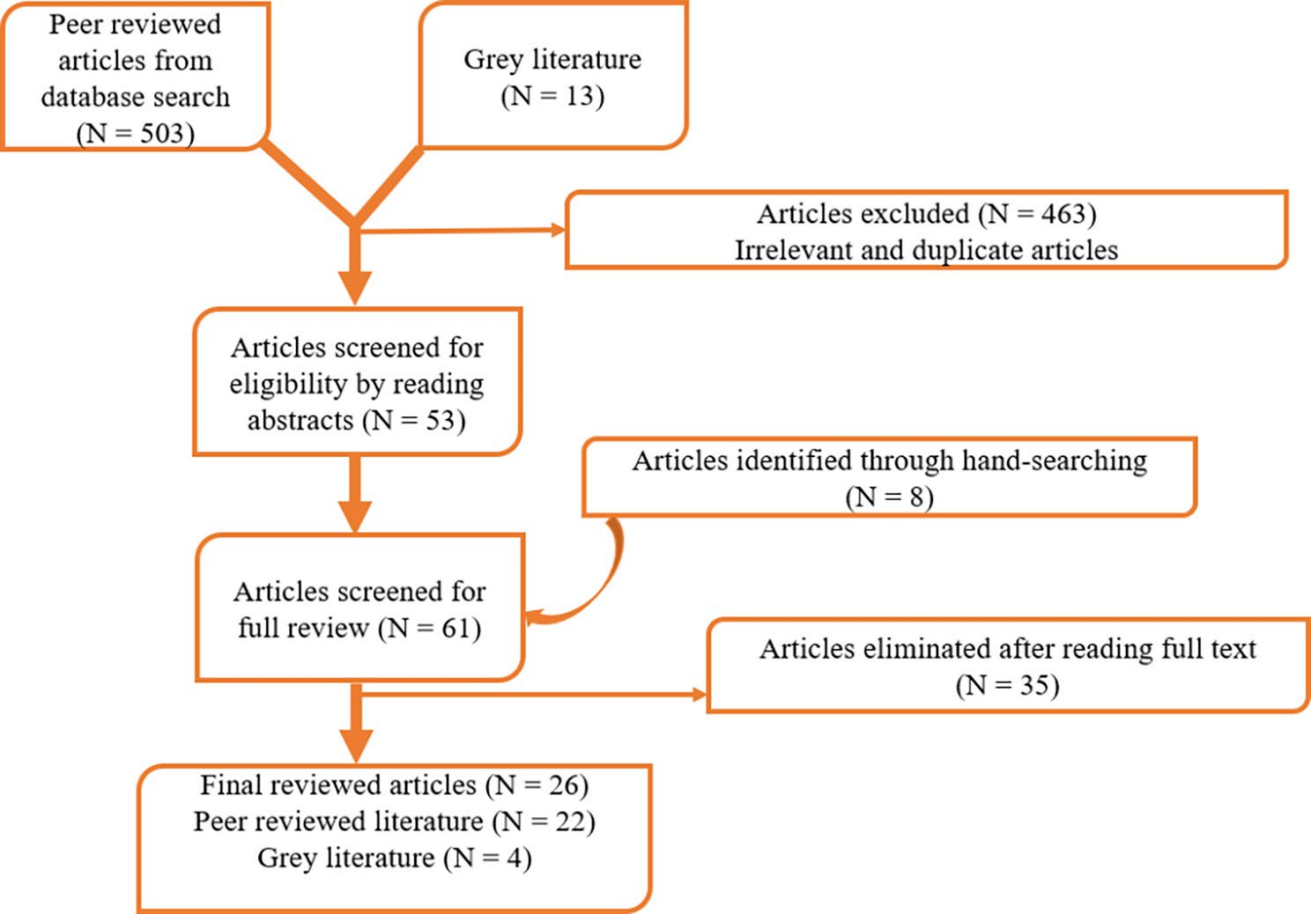
Flow Diagram indicating inclusion and exclusion of articles used for this study

The first step involved the searching of databases (PubMed, Scopus, Popline, Science direct, BioMed Central and google scholar) for peer reviewed articles on the research topic. Grey literature and technical reports were also searched on relevant websites. The second stage involved the exclusion of articles that are not relevant to the topic as well as duplicate articles. In the third stage, the articles left were assessed for eligibility by reading their abstracts. In the fourth stage, hand searching from reference list of included articles was done to retrieve more relevant articles. Next, articles that were not significant for the study were excluded from the fully screened/hand searched articles. In the final stage, peer reviewed articles and grey literature including technical reports that met the specific inclusion criteria were used for the review (Fig. 1).

The quality of the reviewed articles was assessed by authors using an adapted checklist developed by the Joanna Briggs Institute for critical appraisal [14], as described in [15].

### Description of methodological designs of selected articles

Out of the 26 articles that were used for the final review, peer reviewed articles were 22 and 4 were grey literatures. Of the peer reviewed articles, 19 were solely conducted in Ghana and 3 focused on Ghana together with other African countries. Also, out of the peer reviewed articles, 6 were explicitly primary data analysis (qualitative [4] and quantitative [1], mixed method [1]); 7 used explicitly secondary data from the Ghana Demographic Health Survey (GDHS) and regional/district Health and Demographic Surveillance; 2 used secondary data together with qualitative; 3 were review papers. The remaining peer reviewed articles were: policy note [1]; book [1]; and report [2]. The 4 grey articles were mainly reporting, including documents from NHIA, and the World Bank.

## Results

### Presentation of findings

**Fig. 2:**
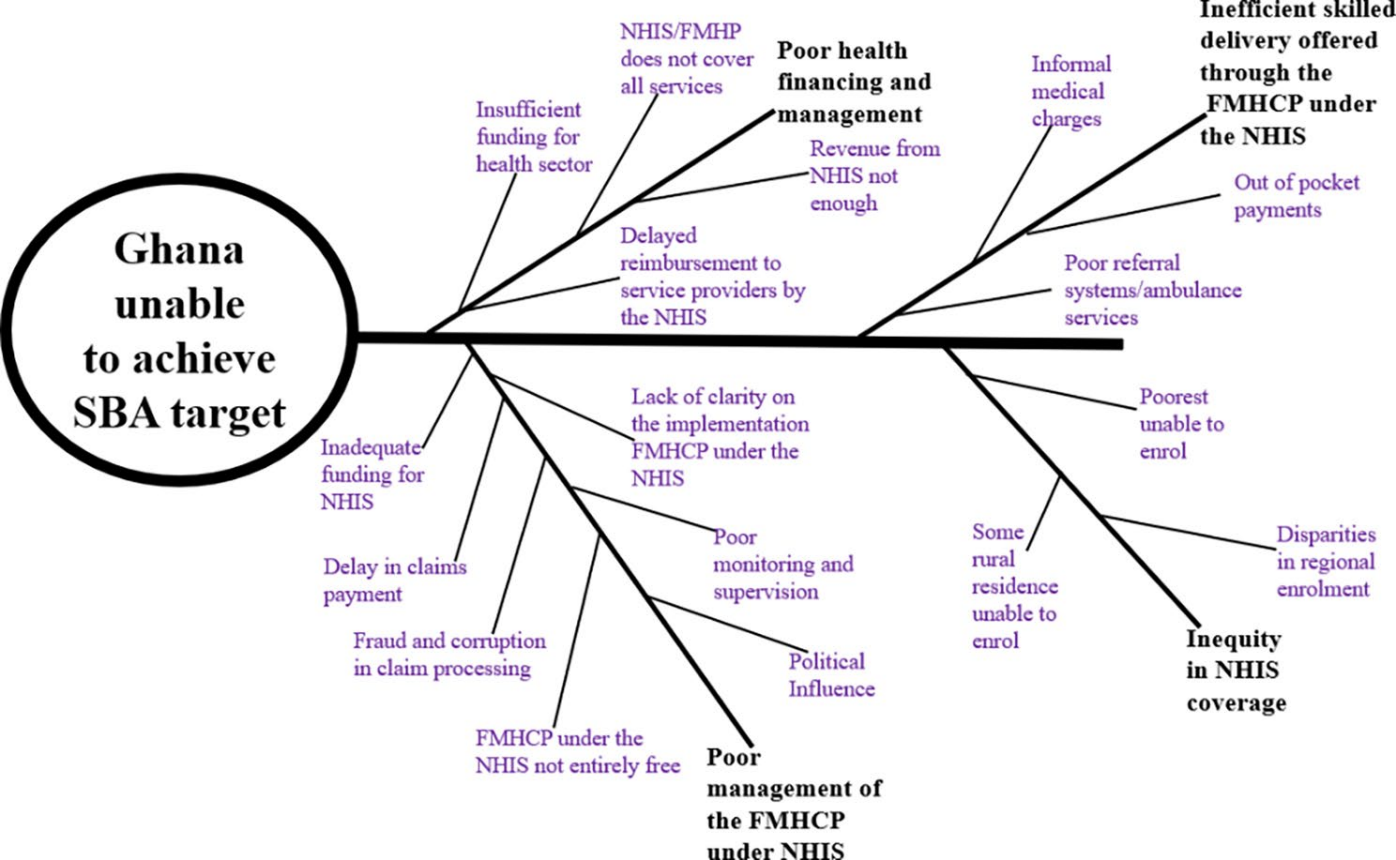
Fishbone analysis of the factors influencing the FMHCP under the NHIS provision for skilled delivery services in Ghana. The inscriptions in bold, have been identified as the major factors influencing the utilization of SBA in Ghana. Although the FMHCP under the NHIS has improved uptake of SBA, some emerging problems like inadequate funding for the policy, poor monitoring and supervision and political influence, are negatively affecting the management of the policy. Also, there is inequity in the coverage of the policy skewed to the disadvantage of the poor and some rural population

A descriptive method was used to present and synthesize the findings from reviewed literature on the factors influencing the FMHCP under the NHIS provision for skilled delivery services in Ghana. Further, a graphical representation of all the findings was presented in a fishbone diagram (Fig. 2).

### The NHIS

#### Coverage of the NHIS and its impact on skilled delivery

##### Inequity in coverage among socioeconomic and demographic groups

There has been an increase in the coverage of the NHIS and utilization of health services in Ghana since the implementation of the NHIS [16, 17]. According to the GDHS 2014, about 62% of respondents (women) mentioned that they were covered by NHIS whereas 38% said they were not covered by any type of insurance. That notwithstanding, inequities still exist among socioeconomic and demographic groups which affect enrolment and wide coverage of the NHIS [16, 18, 19].

NHIS enrolment by members including those entitled to free care such as pregnant women, has seen a substantial growth, however, enrolment is observed to be pro-rich [16, 18, 20, 21]. Subscription onto the NHIS together with other factors like education and marriage all positively contribute to facility delivery [16, 18, 20, 22, 23]. Also, NHIS coverage for maternal health services such as Antenatal Natal Care (ANC) and Post Natal Care (PNC) were significantly associated with uptake of those services. Nevertheless, having insurance coverage for childbirth increased utilization of skilled delivery, though not significantly [18]. This suggests other potential factors influencing skilled delivery services other than insurance ownership.

The possible reason attributed to the enrolment being pro-rich bias, is the subscription fee that is attached to the informal sector members. This may deter them from enrolling unlike with the formal sector members whose premium are being deducted from their pay roll [16].

There are also reported regional differences in enrolment of the NHIS [7, 22, 24] which relate in part to contextual factors such as; the existence of previous NHIS scheme in some regions (for example, Brong Ahafo), population density in urbanized regions making it difficult to enroll a higher population [16] and closeness of health facility [24].

In the findings of Nsiah-Boateng and Aikins [24], increase in new enrolment on the NHIS was observed between the period of 2010 and 2017, particularly in the urban regions such as Ashanti and Greater Accra regions compared to less urbanized regions like UE/R and UW/R.

Furthermore, the coverage of the NHIS by residence among women was 63% for urban and 60.9% (rural), although no significant difference was observed [7].

### Factors influencing enrolment on the NHIS

The perceived factors influencing the enrolment on the policy are: availability of health services, level of education [21, 22], cost of NHIS subscription, convenience of subscription in terms of easy access to the insurance office, and waiting time to collect the cards, influence from peers in terms of their experience with the health insurance, socio-economic status, as well as community beliefs such as; ‘possessing health insurance means buying ill health’ [21].

Meanwhile, a new mobile renewal services was launched in December 2018, to enhance convenience of renewals, reduce the long waiting time of members and promote the scale up of population enrolment on the scheme [25]. Indeed, it was reported by the NHIS that, more than 5 million NHIS subscribers have used the mobile renewal service since its introduction, hence reducing the long queues at the offices [26].

### Implementation and management of the FMHCP under the NHIS

#### Financial sustainability of the policy

The implementation and operation of the NHIS in Ghana is faced with many challenges [10]. The FMHCP under the NHIS was a political agenda which lacked initial costing and budgeting and there were limited stakeholders’ consultation before its operation thus, sustainability has been a major problem [10, 27]. The sustainability of the policy is linked to the general survival of the NHIS. The policy implementers at the district level, lacked adequate information guiding the implementation of the NHIS. The monitoring and evaluation of the policy was also seen to be poor. However, the policy has seen an increase in utilization of services especially for ANC and particularly in areas where women have better access to health services [10].

The NHIS is predominantly funded from these sources; about 72.8% comes from Value Added Tax (VAT), 17.4% from Social Security and National Insurance Trust (SSNIT) contributions from formal sector workers, 4.5% from premiums from informal sector and about 5.3% comes from interest accrued from investments, donor funds, grants and other sources [28]. Individuals employed in the informal sector are those who belong to the non-exempt groups and make direct payments to the scheme for their premiums. These non-exempt groups are required to pay premiums ranging from GH¢ 7.20 to GH¢48.00 based on their income levels. However, since there is often a lack of data on the income level of workers in the informal sector, a flat premium rate is charged during registration process. This flat rate varies from GH¢15.00 in rural areas to GH¢ 22.00 in the urban areas [1]. The limited source of funding which come from premium contributions pose a major barrier in funding the scheme and expanding coverage in the informal sector [28]. Also, the NHIS is perceived to be financially unstable due to the higher number (about 60%) of active members who are under exemption categories such as the under-18s, older people above 70 years, pregnant women and indigents [29].

Furthermore, some design issues of the NHIS that threatens its long-term financial sustainability exist. Normally, insurance is funded by premia because, while membership increases, income from premia would also increase. However, in Ghana over 90% of NHIS funding come from SSNIT and VAT levy [16, 30]. Thus, the growth of its income would be driven by national income growth rather than membership growth [16]. Meanwhile, the growth of membership is higher (33%, 41% and 35% of the population in 2010, 2015 and 2017 respectively) [20], compared to the Gross Domestic Product (GDP) growth (8.5% in 2017) [31]. This implies that, the better the NHIS becomes in terms of providing coverage, the higher the likelihood of facing financial challenges [16]. The revenue generated from the SSNIT and VAT levy are therefore not enough to sustain the NHIS which has greater proportion of membership made up of exempt groups who do not contribute financially to the scheme. Further, the scheme is perceived to be ‘too generous’ as about 95% of treatments are covered [16].

In addition, inadequate funding and poor sustainability of the NHIS results in delays in reimbursement and underfunding of health facilities and this leads to informal medical charges and out of pocket payments for some important services even under the NHIS [16, 27, 32, 33]. Some reasons for the ineffective payment schedules of claims by the scheme are that; the total claims value is usually more than the fund allocated to the scheme from NHIA, which is due to the inclusion of more expensive procedures in the NHIS list. Also, the money generated by the scheme is sometimes used to invest in capital markets or for other purposes, thus makes it difficult to find cash to make timely payments to service providers [34].

### Operational sustainability of the NHIS

#### Provider-centered (supply-side) challenges in the operation of the policy

Although funding and sustainability is a threat to the management of the policy, operational challenges posed much more significant challenge [29] and leads to poor quality of health care offered through the NHIS [35]. In line with this, inefficient processing of claims is one of the internal management problems of the scheme especially with regards to claims vetting [36]. This is because, majority of the claims are vetted manually, making it labor intensive, and as such are subject to errors [27, 36].

More so, the claim vetting system does not provide effective measures for the monitoring of service providers against possible fraudulent behaviors [36]. Corruption and fraudulent practices by claim managers and service providers to extort money from the scheme also exist at facility levels. An example of this is situations where Caesarean Section (C/S) is alleged to have been performed on men. Another example is where about three-quarters of the claims for delivery services are reported to be C/S for a rural setting, which was not possible [37].

Additionally, the introduction of the new payment method for the NHIS referred to as Diagnostic Related Group (DRG) in 2008 has resulted in providers shifting to diagnosis that attract higher tariffs [16]. Attempts have been made to reform the provider payment method by introducing capitation which will help reduce the costs involved in claim processing, enable forecasting of budget by the NHIA and reduce the delays in reimbursement associated with DRG. However, the capitation system is only available at the primary level and for out-patient services only, which makes it difficult to reduce these operational challenges with claim processing [38].

### Client-centered (demand-side) challenges in the operation of the NHIS

There are also concerns about abuse of the scheme by clients referred to as ‘client shopping’, where some NHIS subscribers move from one hospital to the other presenting the same medical complaints just to secure more medicines in order to sell [37, 39]. Some clients also use their friends NHIS card to seek health care services due to poor monitoring and supervision which exists at hospitals [17]. These administrative challenges affect the smooth operation of the policy.

### Political influence in the operation of the NHIS

The National Health Insurance Authority (NHIA), is deemed as an independent regulatory body established to oversee the management of the NHIS, while its regional offices perform monitoring and evaluation roles over the district offices and service providers [39]. This however, is not what exists practically, as political influences pose challenges to the operation and management of the NHIS [16, 39]. In the study of Nsiah-Boateng and Aikins [24], the national level key informants attested to the fact that politics is hindering the smooth collaboration needed to improve enrolment and retention of NHIS members, as well as improve the quality of health service delivery through the NHIS.

### The FMHCP under the NHIS and skilled delivery care in Ghana

Out of pocket payments by pregnant women in health facilities, even with the implementation of the FMHCP under the NHIS, is an issue of concern [40, 41]. In the study of Dalinjong, Wang [40], the total mean cost of out-of-pocket payments made by expectant mothers was estimated at GH¢17.50 [35]. Payments were made for services such as drugs, laboratory, ultrasound scan services as well as informal medical charges for some items needed for delivery. Also, ambulance services for emergency referrals during pregnancy or delivery are not covered under the policy. Women who cannot afford the out-of-pocket payments were deprived of these essential services.

Additionally, there are reported cases of some health service providers extorting monies from clients possessing valid NHIS cards. Monies were extorted ranging from GH¢ 200 to GH¢400 before providing medicines and services such as C/S which were supposed to be covered by the scheme. But these monies were eventually refunded to the victims after a public outcry [42].

Shortages of medicines on the National Health Insurance drug list in health facilities, coupled with the overwhelming workload on service providers, also potentially affects the quality-of-service delivery to clients, including maternity care [24, 40].

## Discussion

This review found that the NHIS, which is meant to be pro-poor, promoting Universal Health Coverage by removing financial barriers to access, as well as improving SBA, has not been able to resolve inequities in enrolment, particularly among the poor and some rural dwellers, despite a general improvement in equity and population coverage of the NHIS. Coverage for free delivery care has many elements and it is necessary that countries choose the most appropriate interventions that will effectively address the inequities in access to SBA. For example, in Morocco a comprehensive free delivery care policy was implemented which also included transportation costs from the home to the health facilities in regions that have poor access. There has been a dramatic improvement for facility and C/S delivery in Morocco, especially among the poorest, over the past 20 years, which is attributed to multiple factors including policy interventions, which addressed both demand and supply side barriers to skilled delivery [43].

This review showed that funding for the NHIS is relatively inadequate to sustain the scheme. Similar to this finding, the study of Witter, Boukhalfa [44] showed that other sub-Saharan African countries such as Mali, Morocco, Benin and Burkina Faso, also rely on domestic sources for funding the policy. However, among these countries the total amount of health spending for the policy in 2011 was 3.5% of total public health expenditure in Burkina Faso, 3% in Benin and 2.5% in Morocco which were potentially sustainable [40].

The financial sustainability of the FMHCP under the NHIS is linked with the structural design of the NHIS and operational challenges, which hinders the effective management of the policy and affects payment of claims to service providers. Ghana’s payment of claims to service providers is done retrospectively, which is the same as what is being practiced in Benin and Burkina Faso, while Morocco pays in advance to service providers on yearly basis in addition to supplying essential medicines and delivery kits [45]. However, unlike Ghana where the payment is done per the NHIS schedule, in Benin for example, the reimbursement was considered over generous and benefited the district health facilities more than the tertiary hospitals, because all facilities were paid a fixed amount - about £ 354 per C/S [45].

These differences in payment of claims show the type of management approaches countries adopt. Unlike most countries where the policy is managed by the government through committees, just like what is being done in Ghana, in Benin, a centralized model was adopted where the management of the policy was being done by independent autonomous agency. This model adopted by Benin has substantially resolved delays associated with the policy [44]. It is evident that there is no single best approach to what works, especially with regards to the management of the policy. However, countries can learn from each other to develop effective policy strategies that can improve access to SBA.

Evidence suggests that, even with the implementation of a comprehensive fee exemption policy, household costs of provision of skilled delivery persist. For example, in Benin and Morocco, users were charged additional fees because service providers wanted to compensate for the free C/S, and all these are due to ineffective monitoring [44]. This is consistent with the findings from this review, where service providers were charging pregnant women for some services that are covered under the policy.

This review revealed that even though skilled birth attendance has improved utilization of skilled delivery, poor women are unable to utilise skilled delivery services compared to their rich counterparts, largely because the NHIS does not fully cover the cost associated with skilled delivery services. This is consistent with another study in Ghana [46].

For an effective free delivery care policy to be successful in removing financial barriers and improving coverage for skilled delivery in Ghana, the government and stake holders involved in the implementation of the policy should increase funding for the policy and ensure its sustainability, as well as addressing the non-financial barriers identified.

### Study limitations

Most articles used secondary data in their analysis, which is limited in obtaining contextual understanding of the findings.

## Conclusions

This review has explored factors influencing NHIS provision for skilled delivery in Ghana. The study showed an improvement in SBA, however, barriers still exist that prevent some women from accessing skilled delivery care. One of such important barriers identified is that informal charges for some services covered by the NHIS persist. Similarly, ambulance services for referring pregnant women during emergencies are not covered under the policy and these challenges hinder access to quality skilled delivery services, especially for lower socioeconomic groups.

The major cause of this, is the inadequate funding and poor sustainability of the NHIS, which results in delays in reimbursement and underfunding of health facilities.

This review therefore proposes that, if Ghana wants to achieve the global target (90%) for SBA as well as the SDG targets 3.1, 3.2 and 3.3.1 by 2030, the NHIA should expand the coverage of the NHIS by absorbing all expenses associated with pregnancy and childbirth for the poor, including the cost of ambulance usage. In addition, the CHPS strategy needs to be strengthened by making the health workforce available, particularly in regions with no access to health services, in order to achieve effective equity in coverage of the policy. Also, the government of Ghana should increase funding for health and the NHIS, to promote the effectiveness and sustainability of the FMHCP under the NHIS in the provision of quality maternal health service.

## Data Availability

All data generated or analysed during this study are included in this published article.

## Abbreviations

SBA: Skilled Birth Attendance
NHIS: National Health Insurance Scheme
FMHCP: Free Maternal Health Care Policy
SDGs: Sustainable Development Goals
MMR: Maternal Mortality Ratio
NMR: Neonatal Mortality Ratio
SBAs: Skilled Birth Attendants
GDHS: Ghana Demographic Health Survey
CHPS: Community-based Health Planning and Services
HIV: Human Immunodeficiency Virus
WHO: World Health Organization
NHIA: National Health Insurance Authority
ANC: Antenatal Natal Care
PNC: Post Natal Care
UHC: Universal Health Coverage
VAT: Value Added Tax
SSNIT: Social Security and National Insurance Trust
DRG: Diagnostic Related Group
C/S: Caesarean Section
Upper East Region: UE/R
Upper West Region: UW/R.
